# Development and validation of a new non-disease-specific survey tool to assess self-reported adherence to medication

**DOI:** 10.3389/fphar.2022.981368

**Published:** 2022-12-07

**Authors:** Rønnaug Eline Larsen, Are Hugo Pripp, Tonje Krogstad, Cecilie Johannessen Landmark, Lene Berge Holm

**Affiliations:** ^1^ Department of Life Sciences and Health, Faculty of Health Sciences, Norway And The Research Group Medicines and Patient Safety, Oslo Metropolitan University, Oslo, Norway; ^2^ Faculty of Health Sciences, Oslo Metropolitan University, Oslo, Norway; ^3^ Department of Biostatistics, Oslo Centre of Biostatistics and Epidemiology, University of Oslo, Oslo, Norway; ^4^ The National Center for Epilepsy, Oslo University Hospital, Oslo, Norway; ^5^ Section for Clinical Pharmacology, Department of Pharmacology, Oslo University Hospital, Oslo, Norway; ^6^ Center for Connected Care, Oslo University Hospital, Oslo, Norway

**Keywords:** non-adherence, measure adherence, assess adherence, patient compliance, reliability, OMAS-37, factor analysis, questionnaire

## Abstract

**Background:** Patients’ non-adherence to medication affects both patients themselves and healthcare systems. Consequences include higher mortality, worsening of disease, patient injuries, and increased healthcare costs. Many existing survey tools for assessing adherence are linked to specific diseases and assessing medication-taking behavior or identifying barriers or beliefs. This study aimed to develop and validate a new non-disease-specific survey tool to assess self-reported medication-taking behavior, barriers, and beliefs in order to quantify the causes of non-adherence and measure adherence.

**Methods:** The survey tool was developed after literature searches and pilot testing. Validation was conducted by assessing the psychometric properties of content, construct, reliability, and feasibility. Content validity was assessed by subject matter experts and construct validity by performing exploratory factor analysis. Reliability assessment was performed by calculating internal consistency, Cronbach’s alpha and test/retest reliability, intraclass correlation coefficient (ICC), and standard error of measurement (SEm). A receiver operating characteristic (ROC) curve and the Lui method were used to calculate the statistical cut-off score for good *versus* poor adherence. Survey responses from Norwegian medication users over 18 years recruited *via* social media were used for the factor analysis and Cronbach’s alpha.

**Results:** The final survey tool contains 37 causes of non-adherence connected to medication-taking behavior and barriers to adherence and beliefs associated with adherence. The overall result for all 37 items demonstrated reliable internal consistency, Cronbach’s alpha = 0.91. The factor analysis identified ten latent variables for 29 items, explaining 61.7% of the variance. Seven of the latent variables showed reliable internal consistency: *medication fear and lack of effect*, *conditional practical issues*, *pregnancy/breastfeeding*, *information issues*, *needlessness*, *lifestyle*, and *avoiding stigmatization* (Cronbach’s alpha = 0.72–0.86). *Shortage* showed low internal consistency (Cronbach’s alpha = 0.59). *Impact issues* and *personal practical issues* showed poor internal consistency (Cronbach’s alpha = 0.51 and 0.48, respectively). The test/retest reliability ICC = 0.89 and SEm = 1.11, indicating good reliability. The statistical cut-off score for good *versus* poor adherence was 10, but the clinical cut-off score was found to be 2.

**Conclusion:** This survey tool, OMAS-37 (OsloMet Adherence to medication Survey tool, 37 items), demonstrated to be a valid and reliable instrument for assessing adherence. Further studies will examine the ability of the tool for measuring adherence enhancing effect following interventions.

## 1 Introduction

Adherence to medications is the process by which patients take their medication as prescribed, comprised of initiation, implementation, and discontinuation (Vrijens et al., 2012). “Increasing the effectiveness of adherence interventions may have a far greater impact on the health of the population than any improvement in specific medical treatments” is an important statement in an influential WHO report from 2003 on medication adherence (Sabaté, 2003). The importance of adherence interventions on patients’ health is still most applicable as failure to adhere is a serious problem affecting both patients and healthcare systems by resulting in higher mortality, worsening of disease, more patient injuries, and increased healthcare costs (Sokol et al., 2005; Cutler et al., 2018; Khan and Socha-Dietrich, 2018; Holbrook et al., 2021; Lu et al., 2021; Majeed et al., 2021; Nymoen et al., 2022).

Adherence rates have an average of around 50% but range widely from 0% to more than 100% (Nieuwlaat et al., 2014; Horne et al., 2019). In 2018, the Organization for Economic Co-operation and Development (OECD) reported that estimates from 2010 suggest non-adherence annually contributes to nearly 2,00,000 premature deaths and costs the European government EUR 125 billion in excess healthcare (Rabia Khan, 2018). In 2004, Norwegian healthcare costs due to incorrect and ineffective medication usage were estimated to be EUR 500 million (Report No. 18 to the Storting, 2004–2005) in a population of 4.6 million people. However, the economic impact of low adherence to medication is difficult to assess due to current research being limited and of mixed quality (Cutler et al., 2018).

The many reasons for non-adherence are thoroughly described in the literature, often showcasing the complexity of adherence behavior (Sabaté, 2003; Hugtenburg et al., 2013; Gast and Mathes, 2019; Horne et al., 2019). One example is the earlier mentioned WHO report, where adherence is viewed as a multidimensional phenomenon determined by the interplay between five different dimensions: patient-related factors, therapy-related factors, social/economic factors, condition-related factors, and health care team and system-related factors (Sabaté, 2003).

It is also widely recognized that non-adherence can be both intentional, e.g., medication deliberately not being taken and/or unintentional, e.g., medication prevented from being taken by barriers beyond one’s own control. Horne et al. have, in this context, displayed the Perceptions and Practicalities Approach (PAPA) (Horne et al., 2019). In PAPA, intentional causes of non-adherence are linked to motivation which depends upon perceptions, e.g., beliefs, emotions, and preferences. Unintentional causes of non-adherence are linked to ability which depends upon practicalities, e.g., capacity, resources, and opportunities. PAPA indicates that adherence is essentially dependent upon individual motivation and ability, which could vary both within and between individuals for different medications and/or timelines. Thus, mapping and quantifying causes for non-adherence are essential in the process of tailoring interventions to enhance adherence.

Patients’ self-reported measures on medication adherence behavior is one of the most common approaches to assess medication adherence (Simoni et al., 2006; Velligan et al., 2006; Paschal et al., 2008; Kelli Stidham Hall et al., 2010; Garfield et al., 2011; Gonzalez and Schneider, 2011; Stirratt et al., 2015). Self-reporting survey tools are often validated by comparing survey data with invasive methods like monitoring drug concentration, blood sugar, blood pressure, and/or cholesterol (Simoni et al., 2006; Velligan et al., 2006; Paschal et al., 2008; Kelli Stidham Hall et al., 2010; Gonzalez and Schneider, 2011). Assessing self-reporting against adequate clinical measurements opens the possibility of predicting clinical outcomes by measuring adherence behavior. Hence, existing self-reporting survey tools are, to a great extent, connected to specific medications and/or medical diagnoses, although there are several different survey tools independent of medication/medical diagnoses (Garfield et al., 2011; Nguyen et al., 2013; Stirratt et al., 2015; Chan et al., 2021) which can be useful, e.g., when assessing non-adherence in general populations. The survey tools differ not only in number of items but, more importantly, also in how these tools map non-adherence. The comprehensive systematic review by Nguyen et al. (2013), which contains the most used validated self-report adherence scales, and the complemented study by Stirratt et al. (2015) are examples of literature showing how adherence scales are focusing either on medication-taking behavior and/or barriers to adherence and/or beliefs associated with adherence. As the PAPA indicates, tailoring interventions are necessary to increase the effectiveness of adherence interventions. One size does not fit all, and adequate knowledge about the causes for non-adherence is vital for tailoring interventions. However, finding an elaborating survey tool that focuses on both medication-taking behavior and barriers to adherence and beliefs associated with adherence has been proven difficult.

Therefore, the aim of this study was to develop and validate a new non-commercial survey tool independent of patients’ medication type and/or medical diagnosis in order to assess self-reported medication-taking behavior, barriers, and beliefs. The overall goal was to make available an adequate tool for measuring adherence and quantifying causes of non-adherence in various patient groups.

## 2 Methods

### 2.1 Development of an online survey tool and questionnaire

The survey tool items are causes of medication-taking behavior, barriers, and beliefs that were identified by literature searches in national (Oria, The Norwegian Electronic Health Library, Norwegian subject libraries, and The Great Norwegian Encyclopedia) and international (PubMed, Google Scholar, and Google) databases. Important search terms were *adherence*, *compliance*, *concordance*, *questionnaire*, *medication*, *self-report*, *patient*, and equivalent terms in Norwegian. The search terms were chosen based on being relevant keywords for existing survey tools for medication adherence.

General recommendations for developing questionnaires were used in the planning and developing phases of the questionnaire (Robson, 2002; Eberhard-Gran and Winther, 2017).

After identifying the items, the items were divided into the five aforementioned WHO dimensions of adherence (Sabaté, 2003). For each item, the medication user was asked “*how often do you not follow the recommendations from your doctor regarding the use of your medication because of* (item)?” Each item was then to be scored on a 4-point Likert scale: “very often”—“often”—“sometimes”—“rarely/never”. The survey tool was built into a questionnaire in Nettskjema (2022). Nettskjema belongs to The University of Oslo and is one of the safest and most used solutions for online data collection for research in Norway.

All of the questions had to be answered to proceed further in the questionnaire, leaving no missing values for completed responses.

Inclusion criteria were Norwegian residents over the age of 18 who had been using medication prescribed and/or recommended by a doctor in the last 12 months. Responders who stated that they were under 18 years, that they had not been using one or more medications prescribed or recommended by a doctor in the last 12 months, or that they were not living in Norway were directed out of the questionnaire before answering the survey tool items.

Responders were also asked demographic questions like gender and education-and to choose from a list of diagnoses to provide information on the ailments for which they had been medicated in the course of the last 12 months. The responders were, in addition, asked a question about their own perception of their overall adherence (see Section 2.4).

Feedback was given on content for the different versions of the survey tool *via* video calls and one-to-one meetings with members of an adherence expert team until there were no more comments from the team.

A few adjustments were made after content validation and feedback given in feasibility pilots (see Section 2.3). A technical verification was performed where the logic of the order of the items was tested after the final version of the survey tool.

### 2.2 Recruitment

For the feasibility pilots, acquaintances of the researchers were invited to participate by answering the online questionnaire and afterward giving feedback on the availability and usability of the online solution, time taken to answer, and clarity of questions and providing suggestions for causes of non-adherence which was not already included.

For the construct validity and internal consistency, Data used were collected as a part of an online survey on medication use. Moderators of several large Norwegian Facebook groups were contacted, and six group moderators replied with consent. An invitation to participate with general information about the study and an electronic link to the questionnaire was then posted on these six Facebook groups. The general invitation addressed group members over 18 years who were using/had been using medication for the last 12 months. To participate, the group members were to use the electronic link and would, in this way, be anonymous. In addition to the survey respondents, data from two pilot studies (not the feasibility pilots) in 2021 using the online questionnaire in Nettskjema were added for the construct validation and internal consistency.

For test/retest reliability: Respondents were recruited from three medium-sized Facebook groups with an invitation to participate anonymously in the test/retest of the questionnaire.

### 2.3 Validation strategy

To make sure survey data are trustworthy, survey tools must be validated—not solely through theoretical constructs but also through empirical constructs. Validity, reliability, and feasibility are important elements of validation. Validity expresses the extent to which an instrument measures what it is designed to measure, and reliability expresses the extent to which outcomes are consistent on repeated measures (Kimberlin and Winterstein, 2008; García de Yébenes Prous et al., 2009; Bolarinwa, 2015). Poor feasibility will influence the response rate and/or interpretation/scoring of survey tool items (García de Yébenes Prous et al., 2009).

Choosing a validation strategy depends on what to measure and if the data fit the assumptions for the selected validation methods (García de Yébenes Prous et al., 2009; Bolarinwa, 2015; McNeish, 2018). The chosen validation strategy is shown in Table 1. Each validation method required an independent population except for construct validity and internal consistency where the same population is used. The population sizes are shown in Table 1 and further explained in the Results-section. Feasibility of the results was tested by piloting.

**TABLE 1:** Validation strategy for the survey tool.

Validation strategy		
Strategies	Methods	n
I FEASIBILITY	Pilots	39
II VALIDITY		
Theoretical construct content validity	Subject matter experts	
Empirical construct validity	Exploratory factor analysis	857
III RELIABILITY		
Internal consistency	Cronbach’s alpha (reliability coefficient)	857
Test/retest reliability	Intraclass correlation coefficient and standard error of measurement	20

Content validity, i.e., to what extent the instrument includes most of the dimensions of the concept being studied (García de Yébenes Prous et al., 2009), was tested by feedback on the online survey tool from the earlier-mentioned adherence expert team on language clarity (wording), completeness, item relevance, and (if any) additional causes of non-adherence.

For construct validity, the exploratory factor analysis (EFA) method of principal axis factoring (PAF) with oblique rotation was performed. Construct validity is to what extent the trait or theory of the phenomenon/concept that the instrument is intended to measure is measured (Bolarinwa, 2015).

For test/retest reliability (consistency across time), the intraclass correlation coefficient (ICC) was calculated for a test/retest group using the survey tool online.

Standard error of measurement (SEm) was calculated using the following formula (Portney and Watkins, 2015): SEm = SD_Test_√(1-ICC) SDTest is the standard deviation of the test.

### 2.4 Measurement of adherence and cut-off score

For each survey tool item, the respondent was asked the following question: “*How often do you not follow the recommendations from your doctor regarding the use of your medication because of* [item]?” For measurement of the adherence score, string value was converted to numeric value: “very often” = 3, “often” = 2, “sometimes” = 1, and rarely/never” = 0, making the total minimum adherence score 0 and maximum adherence score 111.

In order to identify whether the calculated adherence score relates to what the patients believe about their overall adherence, a self-reported adherence question was added to the questionnaire: “*In total, to what extent do you believe you follow the recommendations from your doctor regarding the use of your medication*?” For this anchor question, respondents were to score on a 4-point Likert scale. String value was converted into numeric value for measurement of score: “to a very limited extent” = 4, “to a limited extent” = 3, “to a large extent” = 2, and “to a very large extent” = 1. Thus, indicating that poor adherence would give a higher score, which is in line with the calculated adherence score.

Given a significant correlation, a receiver operating characteristic (ROC) curve was to be made to find the statistical cut-off score for adherence. The ROC curve is a graphical plot illustrating the sensitivity (true positive rate) against the 1-specificity (false positive rate) for various threshold settings—here, the threshold settings being the adherence scores. In order to make the ROC curve, the anchor question scores were dichotomized into whether patients believe they follow the recommendations or not: “to a large extent” and “to a very large extent” = following recommendations = 0, “to a limited extent” and “to a very limited extent” = not following recommendations = 1.

Based on the ROC curve, the Liu method was to be used to calculate the empirical optimal cut point by maximizing the product of the sensitivity and specificity. The empirical optimal cut point would be the statistical cut-off score between good adherence and poor adherence.

All data were analyzed by SPSS Statistics (RRID:SCR_016479) version 27. Empirical optimal cut point was calculated in Stata (RRID:SCR_012763) version 17. The chosen significance level alpha was 0.05.

## 3 Results

### 3.1 Feasibility

Data from three pilots were used for feasibility. The respondents were recruited by three different student groups at Oslo Metropolitan University (OsloMet), and the data were collected in 2021. The three pilots gave complete data from (12 + 15 + 12) 39 online respondents. The respondents first completed the survey tool online and were afterward interviewed by the researchers for feedback on the availability and usability of the online solution, time taken to answer, clarity of questions, and providing suggestions for causes of non-adherence which were not already included. In general, the tested survey tool was feasible, but some feedback was given, especially on the length of some of the items (questions).

The developed survey tool was included in a questionnaire together with sociodemographic and health-related questions. The final questionnaire showed an average responding time of about 10 min for the feasibility pilots.

Just under 80% of the 857 respondents in the survey population used less than 10 min to answer the questionnaire, and over 90% used less than 15 min. Time was measured from the opening of the survey to submitting the survey.

### 3.2 Content validity

Feedback on content validity was given for different adjusted versions of the survey tool *via* video calls and one-to-one meetings with the adherence expert team members until there were no more comments from the adherence expert team. Feedback on content from the feasibility pilots was consecutively included in the adjusted versions of the survey tool.

After the feasibility pilots and the content validation by the adherence expert team, the survey tool ended up containing 37 items connected to medication-taking behavior and barriers to adherence and beliefs associated with adherence.

### 3.3 Construct validity

Completed data from two pilots (n = 121) and the survey group (n = 737) were received, leaving a total of 858 respondents. One respondent scored an unrealistically full score on all 37 items and was thus removed. The calculations were conducted on data from 857 respondents, further referred to as the survey group. Data from the survey group were collected from January to March 2021. The pilot data were collected during the spring of 2021. The demographics of the respondents in the survey group are shown in Table 2.

**TABLE 2:** Demographics of the survey group and test–retest populations.

Demographic profile			
Population		Survey group, n = 857 (100%)	Test–retest, n = 20 (100%)
Age		Range: 18–89	Range: 26–68
	Median	50	51
	Mean	48.3	51.5
	SD Mean	15.3	11
Gender [n (%)]	Female	776 (90.5)	13 (65)
	Male	75 (8.8)	7 (35)
	N/A	6 (0.7)	
Education level [n (%)]	No education	7 (0.8)	
	Primary school only	84 (9.8)	
	High school and the like	446 (52)	8 (40)
	Bachelor’s degree and the like	206 (24)	9 (45)
	Master’s degree and the like	101 (11.8)	3 (15)
	N/A	13 (1.5)	
Chosen diagnosis groups for medication used in the last 12 months [n (%)], multiple choice			
Pain		387 (45.2)	5 (25)
Allergies		309 (36.1)	5 (25)
Cardiovascular diseases		270 (31.5)	6 (30)
Musculoskeletal disorders		253 (29.5)	4 (20)
Sleep-related disorders		223 (26)	3 (15)
Gastrointestinal disorders		207 (24.2)	2 (10)
Psychological disorders		165 (19.3)	1
Lower respiratory tract diseases		152 (17.7)	2 (10)
Endocrine diseases		131 (15.3)	5 (25)
Dermatological disorders		120 (14)	1 (5)
Gynecological disorders and contraception		98 (11.4)	1 (5)
Upper respiratory tract and otorhinolaryngologic disorders		93 (10.9)	2 (10)
Fever, nausea, vomiting, dizziness, travel and motion sickness, hiccups, restless legs, leg cramps, etc.		86 (10)	1 (5)
Infectious diseases		80 (9.3)	
Immune system malfunctions and transplants		69 (8.1)	
Other		68 (7.9)	
Nervous system diseases		39 (4.6)	1 (5)
Kidney and urinary tract disorders		35 (4.1)	1 (5)
Blood-related disorders		34 (4)	
Palliative care		31 (3.6)	
Eye disorders and diseases		24 (2.8)	
Cancer		18 (2.1)	1 (5)
Obstetrical disorders		10 (1.2)	
Prostate problems		4 (0.5)	1 (5)
Substance abuse problems		2 (0.2)	
Do not know/do not want to tell/not applicable		2 (0.2)	

Pearson correlation was calculated to measure the strength of the linear variables as linear correlation is an assumption for factor analysis. 1,230 of the 1,332 variables showed a significant (*p* ≤ 0.05) linear correlation.

Kaiser–Meyer–Olikin (KMO) measure of sampling adequacy was performed to see if the correlations between the variables were fit for factor analysis. KMO for all items in total was 0.89. A total of 30 items had KMO over 0.8, and seven items had KMO between 0.79–0.61 (see Table 3). Since the KMO measure for all of the items was over 0.6, the data were fit for factor analysis. This is supported by Bartlett’s test of sphericity being significant (*p* ≤ 0.05).

**TABLE 3:** Kaiser-Meyer-Olkin (KMO) values for each survey item.

Items	KMO
All 37 items in total	0.89
You do not want to be sick, and taking medication is a reminder of this	0.95
You are fearing getting addicted to the medication	0.95
Financial reasons	0.93
You have used the same type of medication before without them having good/satisfactory effect	0.93
You are using many drugs simultaneously	0.92
You are not feeling any effect of the medication	0.92
You are, in principle, against medication treatment	0.92
You are feeling more sick taking them	0.91
You are feeling stigmatized or made sick by having to use medication	0.90
You cannot stand taking medication	0.90
You reckon it does not matter using the medication or not	0.90
You do not feel sick	0.90
You prefer alternative treatment	0.89
You are feeling better	0.89
You are fearing adverse effects	0.89
You are feeling clever when using less than recommended by the doctor	0.89
You feel medications are harmful, toxic and/or you do not tolerant them	0.88
It does not suit your lifestyle to use medication	0.88
You do not want others to know that you are using medication	0.87
Little or no information from the doctor, pharmacy, or other health personnel on how to use your medication	0.87
You do not want to go to the pharmacy due to the corona pandemic	0.87
You have difficulties in taking medication due to specific instructions (like with and without food, in an upright position *etc.*)	0.86
Need of driving a car	0.86
You have difficulties in taking medication at specific hours	0.85
Practical reasons (such as difficulty in opening the packaging, pushing tablets out of the blister packaging, or splitting/crushing the tablet)	0.85
The medications were sold out or not available at the pharmacy	0.85
Misunderstandings related to generic medication (medication with the same content but from different manufacturers)	0.84
You are being influenced by media, the internet, friends, family, and/or others	0.83
You forgot to take the medication	0.82
You are out of medication	0.82
Ethical or religious reasons	0.79
You have difficulties in accessing a pharmacy	0.75
Disabilities (like difficulty in swallowing the tablet or impaired vision making finding the right medication difficult)	0.74
You forgot how to use them	0.69
You did not understand what the doctor or pharmacy staff meant	0.69
You are breastfeeding	0.63
You are pregnant	0.61

EFA was performed to find clusters of inter-correlated variables, so-called latent variables or factors. PAF with oblique (Oblimin) rotation extracted ten latent variables with eigenvalue >1, explaining a total of 61.7% of the variance (see Table 4). An acceptable variance explained for the construct to be valid is said to be more than 60% in factor analysis (Hair, 2014). Table 5 shows the pattern matrix for the ten latent factors with 29 associated item-loadings > +/- 0.4. The remaining eight of the 37 items did not show loadings > +/- 0.4. Rotation converged in 14 iterations.

**TABLE 4:** Validation values for factors and items.

Items	Eigenvalue	% of variance	Cronbach’s *α*	Corrected item–total correlation–all items	Corrected item–total correlation–interfactoral
All 37 items			0.91		
Factor 1: Medication fear and lack of effect	9.06	24.48	0.78		
You are fearing getting adverse effects				0.63	0.63
You feel medications are harmful, toxic and/or you do not tolerant them				0.69	0.66
You have used the same type of medication before without them having good/satisfactory effect				0.58	0.53
You are not feeling any effect of the medication				0.61	0.54
Factor 2: Conditional practicalities	2.33	6.32	0.72		
You have difficulties taking the medication at specific hours				0.51	0.69
You have difficulties taking medication due to specific instructions (like with and without food, in an upright position *etc.*)				0.47	0.52
You forgot				0.35	0.46
Factor 3: Pregnancy/breastfeeding	1.89	5.11	0.86		
You are pregnant				0.21	0.75
You are breastfeeding				0.24	0.75
Factor 4: Information issues	1.76	4.76	0.78		
You forgot how to use them				0.25	0.64
You did not understand what the doctor or pharmacy staff meant				0.27	0.64
Factor 5: Needlessness	1.63	4.41	0.74		
You reckon it does not matter using the medication or not				0.46	0.49
You are feeling better				0.55	0.64
You do not feel sick				0.55	0.59
Factor 6: Shortage	1.49	4.03	0.58		
Financial reasons				0.51	0.38
The medications were sold out or not available at the pharmacy				0.28	0.39
You are out of medication				0.32	0.42
Factor 7: Avoiding stigmatization	1.32	3.57	0.74		
You do not want others to know that you are using medication				0.48	0.57
You are feeling stigmatized or made sick by having to use medication				0.55	0.62
You are feeling clever when using less than recommended by the doctor				0.45	0.42
You do not want to be sick, and taking medication is a reminder of this				0.60	0.54
Factor 8: Lifestyle	1.18	3.19	0.72		
It does not suit your lifestyle to use medication				0.49	0.59
You prefer alternative treatment				0.49	0.60
You are, in principle, against medication treatment				0.53	0.60
Ethical or religious reasons				0.31	0.38
Factor 9: Impact issues	1.15	3.10	0.51		
You are being influenced by media, the internet, friends, family, and/or others				0.31	0.35
You have difficulties accessing a pharmacy				0.25	0.35
Factor 10: Personal practicalities	1.02	2.75	0.48		
Practical reasons (such as difficulty in opening the packaging, pushing tablets out of the blister packaging, or splitting/crushing the tablet)				0.37	0.33
Disabilities (such as difficulty in swallowing the tablet or impaired vision making finding the right medication difficult)				0.20	0.33
Items with loadings ≤0.4					
You do not want to go to the pharmacy due to the corona pandemic				0.36	
Need of driving a car				0.31	
You are fearing getting addicted to the medication				0.60	
You are using many drugs simultaneously				0.43	
You are feeling more sick taking them				0.58	
You cannot stand taking medication				0.59	
Little or no information from the doctor, pharmacy, or other health personnel on how to use your medication				0.43	
Misunderstandings related to generic medication (medication with the same content but from different manufacturers)				0.34	

**TABLE 5:** Pattern matrix for PAF extraction, oblimin with Kaiser normalization rotation and loading > +/−0.4.

Items	Factors									
	1 Medication fear and lack of effect	2 Conditional practicalities	3 Pregnancy/breastfeeding	4 Information issues	5 Needlessness	6 Shortage	7 Avoiding stigmatization	8 Lifestyle	9 Impact issues	10 Personal Practicalities
You feel medications are harmful, toxic and/or you do not tolerant them	0.62									
You are fearing adverse effects	0.47									
You have used the same type of medication before without them having good/satisfactory effect	0.42									
You are not feeling any effect of the medication	0.41									
You have difficulties taking the medication at specific hours		0.72								
You have difficulties taking medication due to specific instructions (such as with and without food, in an upright position, *etc.*)		0.54								
You forgot		0.52								
You are breastfeeding			0.96							
You are pregnant			0.81							
You did not understand what the doctor or pharmacy staff meant				0.80						
You forgot how to use them				0.76						
You are feeling better					0.74					
You do not feel sick					0.65					
You reckon it does not matter using the medication or not					0.51					
You are out of medication						0.51				
The medications were sold out or not available at the pharmacy						0.50				
Financial reasons						0.46				
You do not want others to know that you are using medication							0.68			
You are feeling stigmatized or made sick by having to use medication							0.57			
You do not want to be sick, and taking medication is a reminder of this							0.41			
You are feeling clever when using less than recommended by the doctor							0.40			
It does not suit your lifestyle to use medication								0.66		
Ethical or religious reasons								0.57		
You are, in principle, against medication treatment								0.46		
You prefer alternative treatment								0.46		
You are being influenced by media, the internet, friends, family, and/or others									0.72	
You have difficulties accessing a pharmacy									0.43	
Practical reasons (like difficulty in opening the packaging, pushing tablets out of the blister packaging, or splitting/crushing the tablet)										0.58
Disabilities (such as difficulty in swallowing the tablet or impaired vision making finding the right medication difficult)										0.54

Factor 1 encompasses almost 25% of the total variance and includes four items, where two items describe fear of medication outcomes (adverse effects and non-tolerance) and two items describe lack of effect. See Table 4 for % of variances. Factor 2 encompasses over 6% of the variance containing three items regarding conditional practicalities like forgetting and difficulties taking the medication due to timing and/or specific instructions. Factor 3 encompasses 5.1% of the variance and includes the two items directly connected to pregnancy and breastfeeding. Factors 4–10 encompass variances between 4.8 and 2.8%. Factor 4 connects the information issues of not understanding what the doctor/pharmacy staff meant and forgetting how to use the medication. Factor 5 includes three items describing no need for medication, like feeling better, not feeling sick, and thinking that it does not matter whether the medication is used or not. The three items on Factor 6 involve shortage issues like having no medication left, lack of availability in the pharmacy, and financial reasons. The four items of factor 7 are connected to wanting to avoid stigmatization. Two items are about not wanting to be sick, where medication is a reminder that stigmatizes, and two items are about feeling clever when taking less than prescribed and not wanting others to know about the medication. Factor 8 involves four lifestyle issues: ethical/religious reasons, preferring alternative treatments, being in principle against medication treatment, and belief that taking medication does not suit the lifestyle. Factor 9 connects the impact of being influenced by media, the internet, friends, family, and others to the difficulties of accessing a pharmacy. Factor 10 is the last factor and embraces two items regarding personal practicalities of handling the medication.

### 3.4 Reliability

#### 3.4.1 Internal consistency

The data from the 857 respondents in the survey group used for construct validity were also used for internal consistency.

Cronbach’s *α* was calculated for internal consistency. The overall result for all 37 items in total demonstrated a very reliable internal consistency with Cronbach’s *α* 0.91 (See Table 4). Factor 1–5 and 7–8 showed reliable internal consistency with Cronbach’s *α* between 0.72–0.86. Factor 6 showed low reliable internal consistency with Cronbach’s *α* = 0.58, and Factors 9 and 10 had poor reliable consistency with Cronbach’s *α* = 0.51 and 0.48, respectively. Although factors 6, 9, and 10 *per se* showed low/poor reliability, removal of either of the factor items had no particular impact on the overall Cronbach’s *α* of 0.91.

Exploratory factor analysis was chosen to explore latent variables and not to remove eventual redundant items. Eight of the items had loadings < +/− 0.4 and were thus not included in the factors. Removal of any of these items had no particular impact on the overall Cronbach’s *α* of 0.91.

The corrected item–total correlation values for the items indicate overall good discrimination between all 37 items and between the items in each factor as all values exceeded 0.2 (See Table 4).

#### 3.4.2 Test/retest reliability

Data were collected during the first half of 2022, with 14 days between publishing the web link for the test and the retest.

A total of 47 responded to the test, and 22 of these responded to the retest. Two were removed due to answering the test and the retest being too close apart (<7 days), leaving 20 respondents and a response rate of 42.5%. The 20 respondents answered the test and the retest with a median interval of 13 days apart (range: 8–24 days).

The average measure was ICC = 0.89 and SEm = 1.11, both indicating good reliability (Matheson, 2019). ICC was calculated using a two-way random model and absolute agreement, and SEm using the test standard deviation (SD).

#### 3.4.3 Measurement of adherence and cut-off score

Data from three of the 857 respondents were excluded as they answered “Do not know/not applicable/do not want to answer” on the anchor question, leaving n = 854. The linear regression analysis on the anchor question toward the adherence scores showed a significant correlation (*p* ≤ 0.05) between the two measures of adherence with an acceptable R-squared = 0.24.

**FIGURE 1:**
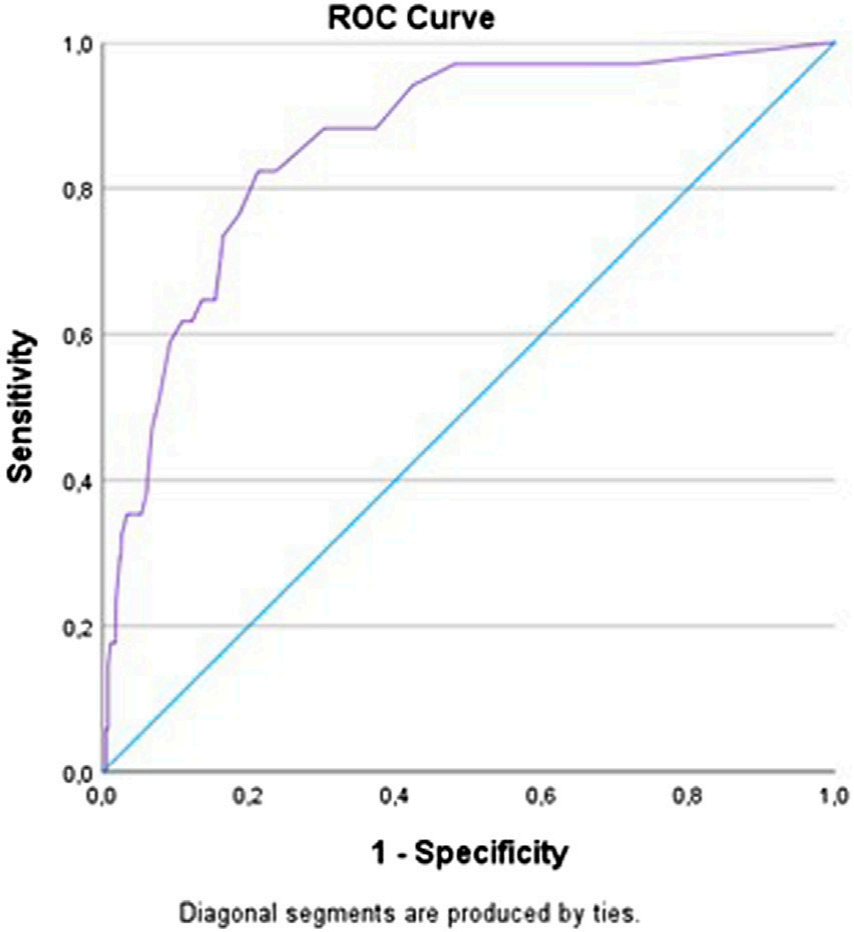
ROC curve for anchor question *versus* adherence score. The ROC curve is produced in SPSS.

The dichotomization of the anchor question into whether the patients believe they follow the recommendations or not resulted in n = 820 for the group that believes they follow (values for “to a large extent” and “to a very large extent”) and n = 34 for the group that does not believe they follow (values for “to a small extent” and “to a very small extent”). The ROC curve based on this dichotomization of the anchor question is shown in Figure 1. The area under the curve (AUC) shows a significant (*p* ≤ 0.05) high classification accuracy value of 0.86. The empirical optimal cut point for the adherence score scale was 10 (sensitivity = 0.82, specificity = 0.79, and AUC = 0.81), leaving the statistical cut-off score for adherence to be 10.

## 4 Discussion

This study was conducted to develop a survey tool that measures adherence and quantifies causes of non-adherence independently of patients’ medication type and/or medical diagnosis and to evaluate the psychometric properties and factor structure of the survey tool. As mentioned in Section 1, it has been proven difficult to find an elaborating survey tool that focuses on both medication-taking behavior and barriers to adherence and beliefs associated with adherence. The importance of assessing behavior, barriers, and beliefs is imperative when tailoring interventions for non-adherence and is the main rationale for developing this survey tool.

### 4.1 Development and validation

The overall result for all 37 items of the survey tool demonstrated a very reliable internal consistency with Cronbach’s *α* 0.91. Cronbach’s *α* is sensitive to the number of items, and some literature suggest that *α* should not exceed 0.9. If *α* exceeds 0.9, it may suggest that some items are testing the same but from a different angle and should be removed (Tavakol and Dennick, 2011). In our study, the *α* is approximately 0.9, the removal of any items had no particular impact on the overall *α*, and the corrected item–total correlation values for all of the 37 items indicated good discrimination. When quantifying causes of non-adherence, it is important to cover all well-known issues and the calculations on internal consistency support keeping all of the 37 items.

EFA was chosen for construct validity to explore underlying factor structures. PAF extracted ten latent factors with eigenvalue >1. Most of the latent factor dimensions are all well-known and showed reliable internal consistency: *conditional practicalities* (Factor 2), *being pregnant/breastfeeding* (Factor 3), *needlessness for medication* (Factor 5), *wanting to avoid stigmatization* (Factor 7), and *lifestyle issues* (Factor 8). However, the latent dimension of *medication fear combined with lack of effect* (Factor 1) was interesting and should be further investigated. It is also interesting to unravel that it is not necessarily lack of information on how to use medication that makes people forget how to use them, but rather that they do not understand the explanations from the doctor or pharmacy staff, *information issues* (Factor 4). The *shortage* (Factor 6) showed low reliable internal consistency even though the combination of issues could be expected, and removal of any of the three items did not improve the *α*. The *impact issue* (Factor 9), which is a combination of being influenced by media, the internet, friends, family, and/or others, and difficulties in accessing a pharmacy was unforeseen, and the poor reliable consistency was to be expected. The *personal practicalities* (Factor 10) combination also showed poor reliable consistency even though the combination was expected. This could be explained by the low number of respondents choosing options other than “rarely/ never” for these two items (56 and 28, respectively).

The survey tool items are divided into the five WHO dimensions (Sabaté, 2003): patient-related factors, therapy-related factors, social/economic factors, condition-related factors, and health system/HCT factors. There were, however, some difficulties in placing the 37 items between the five dimensions as several of the items could fit into more than one dimension. Exchanging the WHO dimensions with latent variable dimensions from the performed EFA would be interesting to investigate further.

The average measure of ICC and SEm indicated both good test/retest reliability. The 20 respondents replied to the test and retest with an interval of 8–24 days with a median interval of 13 days apart. In the literature, there is a wide range of administration intervals used in test/retesting depending, e.g., upon assessment of the stability of the condition involved and complexity of the patient-reported outcome (Quadri et al., 2013). For this study, the medication condition could change over time, and the time frame should not be too long. The interval should, however, be long enough to not remember the test answers when taking the retest. It was thus decided to analyze the respondents who had replied between 1–4 weeks. Although the average measure of ICC and SEm showed good test/retest reliability the sample size of 20 might be a bit low (Terwee et al., 2012).

### 4.2 Measurement of adherence and cut-off score

The survey tool aims to measure adherence. For every item, the respondent is to score “very often”—“often”—“sometimes”—“rarely/never” on the question “*How often do you not follow the recommendations from your doctor regarding the use of your medication because of [item]*?*”* Every item will weigh equal as the clinical outcome of the non-adherence will be the same, i.e., if the respondent scores “very often,” it does not matter if not taking the medication very often is because of forgetting to take the medication or being influenced by others *etc.* But not every item is of relevance for everyone, e.g., items regarding pregnancy and breastfeeding. This is why the scores are converted from string to numeric value, and adherence is measured by the total numeric adherence score.

Clinically it would be considered as poor adherence if the patient “often” (2 points) or “very often” (3 points) does not follow the recommendations for one reason, and it could also be considered as poor adherence if the patient “sometimes” (1 point) does not follow the recommendations for several reasons. This indicates that an adherence score ≥2 could be considered poor adherence, whereas an adherence score of 1 or 0 could be considered good adherence.

The correlation between the adherence score and the anchor question *“in total, to what extent do you believe you follow the recommendations from your doctor regarding the use of your medication*?” were significant (*p* ≤ 0.05), and the AUC of the ROC curve showed high classification accuracy. If one considers the anchor question to be the truth (or the respondent’s claimed truth), this demonstrates that the adherence score is a good measure of the degree of adherence. The statistical cut-off score for adherence was calculated to be 10 based on ROC. Even though the anchor question and the adherence score showed a significant correlation, the statistical calculated cut-off score for adherence could not be used clinically. The respondents that scored between the clinical cut-off for adherence of two and the statistical calculated cut-off score of 10 believed they were following the doctor’s recommendation although they, in fact, did not, showing an overestimation of adherence score. This supports the knowledge of self-reporting as subject to social-desirability biases (Kimberlin and Winterstein, 2008; Stirratt et al., 2015).

### 4.3 Limitations

This study used the 4-point Likert rating scale for both the adherence score questions and the anchor question. Much research has been carried out without reaching an agreement regarding finding the optimal number of response categories for Likert scales in order to maximize the scales’ psychometric properties (Chang, 1994; Xu and Leung, 2018; Taherdoost, 2019). The 4-point Likert scale is a forced scale because of the lack of neutral options and was chosen to force the respondent to form an opinion of the items. Larger numbers of even Likert scales could have been chosen, but this could go beyond the discrimination abilities of respondents and create indistinct measurements. However, it has been indicated that the 4-point scale could have higher skewness and lower loadings than a larger number of Likert scales (Xu and Leung, 2018).

Self-reporting is subject to challenges with social-desirability biases (Kimberlin and Winterstein, 2008; Stirratt et al., 2015), meaning that respondents are answering in a way where they are well-presented in the eyes of others which does not necessarily reflect the reality. For each survey tool item, the respondent was asked: “*how often do you not follow the recommendations from your physician regarding the usage of your medication because of* [item]?” This approach in the questioning was chosen to reassure the patient from feeling shame for not adhering to medication by demonstrating various known causes for non-adherence and thus opting for a more honest scoring.

The performed validations do not include concurrent validity. Due to structural differences in sample strategy, sample size, and population, the correlated measures comparing studies can be challenging (Garfield et al., 2011). However, this should be investigated further when assessing findings after the use of this new survey tool.

For the content validation the adherence expert team did not utilize any scale measurement making the content validation process less documented and with no possibility of calculating a content validity index (CVI).

Recruitment was done *via* Facebook in an attempt to get many respondents. A systematic review from 2017 (Whitaker et al., 2017) states growing evidence for Facebook being a useful recruitment tool for health research due to, e.g., shorter recruitment period and easier to access demographics that are hard to reach. However, one limitation is internet accessibility—seniors aged 65 + being the smallest demographic group on Facebook (only 4.8%) (OMNICORE, 2022). The age distribution in our study (see Table 2) reflects this and can indicate age bias.

Another bias is that females are more likely to respond to surveys (Smith, 2008). This is also applicable to our study as 90.4% of the respondents were females (see Table 2), although Facebook is used by more males (56%) than females (44%) (OMNICORE, 2022).

There is also a bias of educated people being more likely to participate in surveys than less educated people (Smith, 2008). The survey tool was piloted and validated in the Norwegian language only. In our study, 10.6% of the responders were below upper secondary education, and 35.8% had higher education (see Table 2). Norwegian statistics from 2020 show that 24.8% of the population are below upper secondary education, and 35.3% have higher education (SSB, 2020), demonstrating that our respondents, in total, had more education than the general population in Norway.

The response rate was not possible to calculate for construct validity. The participants were recruited by Facebook groups, so it is not possible to know how many of the group members actually saw the invitation nor how many of the group members were relevant for the questionnaire (over 18 years, using medication, or had used medication for the last 12 months).

The survey tool contains three double-barred questions: *you do not want to be sick and taking medication is a reminder of this*/*you are feeling stigmatized or made sick by having to use medication*/*you feel medications are harmful, toxic and/or you do not tolerate them*. To avoid misconceptions in newer versions, these should be changed into the following: *taking medication is a reminder of being sick*/*you are feeling stigmatized by having to use medication*/*you feel medications are doing you more harm than good*.

The validated survey tool is named OMAS-37 (OsloMet Adherence to medication Survey tool, 37 items).

## Conclusion

This study describes the development and validation of a self-reporting adherence survey tool (OMAS-37) where causes for non-adherence are quantified, and adherence is measured. The validated survey tool is named OMAS-37 (OsloMet Adherence to medication Survey tool, 37 items). The OMAS-37 demonstrated to be a valid and reliable instrument. The OMAS-37 is, to our knowledge, the first non-disease-specific adherence instrument developed to assess self-reported causes of medication-taking behavior, barriers, and beliefs. Further studies will examine the ability of the tool for measuring adherence enhancing effect following interventions.

## Data Availability

The raw data supporting the conclusions of this article will be made available by the authors, without undue reservation.
